# Practicability and Diagnostic Yield of One-Stop Stroke CT with Delayed-Phase Cardiac CT in Detecting Major Cardioembolic Sources of Acute Ischemic Stroke: A Proof of Concept Study

**DOI:** 10.1007/s00062-021-01003-7

**Published:** 2021-03-10

**Authors:** Friederike Austein, Matthias Eden, Jakob Engel, Annett Lebenatus, Naomi Larsen, Marcus Both, Tim-Christian Piesch, Mona Salehi Ravesh, Johannes Meyne, Olav Jansen, Patrick Langguth

**Affiliations:** 1 Department of Neuroradiological Intervention and Diagnostics, University Hospital Hamburg-Eppendorf, Martinistr. 52, 20251 Hamburg, Germany; 2 Department for Radiology and Neuroradiology, University Hospital Schleswig-Holstein, Campus Kiel, Kiel, Germany; 3 Department for Internal Medicine III, Molecular Cardiology and Angiology, University Hospital Schleswig-Holstein, Campus Kiel, Kiel, Germany; 4 Department for Neurology, University Hospital Schleswig-Holstein, Campus Kiel, Kiel, Germany

**Keywords:** Imaging, Multislice computed tomography, Transthoracic echocardiography, Performance, Cardioembolic stroke

## Abstract

**Purpose:**

Recurrent stroke is considered to increase the incidence of severe disability and death. For correct risk assessment and patient management it is essential to identify the origin of stroke at an early stage. Transthoracic echocardiography (TTE) is the initial standard of care for evaluating patients in whom a cardioembolic source of stroke (CES) is suspected but its diagnostic capability is limited. Transesophageal echocardiography (TEE) is considered as gold standard; however, this approach is time consuming, semi-invasive and not always feasible. We hypothesized that adding a delayed-phase cardiac computed tomography (cCT) to initial multimodal CT might represent a valid alternative to routine clinical echocardiographic work-up.

**Material and Methods:**

Patients with suspected acute cardioembolic stroke verified by initial multimodal CT and subsequently examined with cCT were included. The cCT was evaluated for presence of major CES and compared to routine clinical echocardiographic work-up.

**Results:**

In all, 102 patients with suspected acute CES underwent cCT. Among them 60 patients underwent routine work-up with echocardiography (50 TTE and only 10 TEE). By cCT 10/60 (16.7%) major CES were detected but only 4 (6.7%) were identified by echocardiography. All CES observed by echocardiography were also detected by cCT. In 8 of 36 patients in whom echocardiography was not performed cCT also revealed a major CES.

**Conclusion:**

These preliminary results show the potential diagnostic yield of delayed-phase cCT to detect major CES and therefore could accelerate decision-making to prevent recurrence stroke. To confirm these results larger studies with TEE as the reference standard and also compared to TTE would be necessary.

## Introduction

Today, patients presenting at the emergency department (ED) with symptoms of acute ischemic stroke (AIS) due to large-vessel occlusion are typically examined with multimodal computed tomography (CT), which includes noncontrast-enhanced CT, CT angiography (CTA), and CT perfusion (CTP) [1]. Discovering potential cardiac sources of stroke represents an important part of the emergency evaluation of AIS patients as it often impacts treatment decisions that are essential for determining secondary stroke prevention strategies; however, the optimal approach for cardiac work-up in AIS patients is not known [2]. Echocardiographic evaluation is recommended in the acute phase of stroke mainly for etiological and therapeutic purposes but also for assessing cardiovascular comorbidities; however, facing an increasing incidence of stroke, particularly concerning access to transesophageal echocardiography (TEE) owing to organizational strains, there are often delays in completing the examinations during the hospital stay, which increases the length of hospitalization [3, 4] and thus contributes to an increase in both direct and indirect healthcare costs. Indeed, variations and delays in diagnostic procedures during hospitalization after AIS are common [5–7].

The use of CT imaging of the heart has shown a high sensitivity and specificity for detecting sources of cardioembolic stroke (CES) [8, 9]. We hypothesized, therefore, that adding a delayed-phase cardiac CT (cCT) to the initial multimodal CT might represent a valid alternative to routine clinical echocardiographic (Transthoracic Echocardiography [TTE] or TEE) work-up.

## Material and Methods

### Patient Selection

This retrospective study was performed according to the protocol (D 567/18) approved by the institutional ethics committee of Christian-Albrechts University Kiel and informed consent was waived due to the retrospective nature of this study. The hospital database was screened for all patients with AIS verified by multimodal CT between September 2018 and August 2019 and in whom delayed-phase cCT images were additionally acquired because a cardioembolic origin of stroke was suspected.

### CT Examination

All CT examinations were performed on a 128-section multidetector CT system (iCT; Philips Healthcare, Best, The Netherlands) without administering heart rate-reducing agents to patients beforehand. The acquisition parameters for the additional heart protocol for adults were as follows: 64 × 0.625mm collimation, 0.27s gantry rotation time, 100kV or 120kV tube voltage, and 375mA tube current.

The multimodal CT combined with a delayed-phase cardiac CT protocol included the following sections: a) anteroposterior and lateral scout view, b) noncontrast-enhanced brain CT, c) nonelectrocardiography (ECG)-gated CTA ascending from the origin of the aortic arch to the vertex of the head, d) CTP of the brain and e) delayed heart CT covering the entire heart, performed during a single breath-hold and ECG-gated retrospectively, descending from the origin of the aortic arch to the diaphragm.

The contrast agent dose (Imeron 350, Bracco, Milan, Italy) consisted of 40 ml for CTA, 40 ml for CTP, and 45 ml for the cardiac CT, giving a total contrast agent dose volume of 125 ml. Cardiac imaging was performed immediately after CTA and CTP, injecting the contrast agent at a rate of 5 ml/s.

### Radiation Dose for CT Protocol

The radiation dose was monitored in the patients for each study by standard dose indicator dose length product (DLP). The CT system calculated DLP for each CT study and automatically saved it to a dose report.

### Image Analysis

The results of all stroke and cardiac examinations were analyzed in a final expert consensus reading. All stroke examinations were analyzed by two independent neuroradiologists (N.L., F.A.) who have at least 10 years and 6 years experience, respectively, and the cardiac examinations were analyzed by two cardiovascular radiologists (P.L., T.P.) with at least 6 years and 4 years experience, respectively. All examiners were blinded to the clinical findings and other results. For neuroradiology imaging the stroke location and for cardiac imaging the major CES were reported. Of note, minor cardiac sources such as interatrial septal aneurysm and patent foramen ovale (PFO) were not analyzed in this study. In cCT, a thrombus was defined when the filling defect was oval or round. A triangular, less contrasting defect in the left atrial appendage (LAA) was considered as a circulatory disorder.

Imaging quality of the CT examinations was graded into three categories (good, moderate, and poor).

### Echocardiographic Examination

All echocardiographic examinations were routinely performed by experienced examiners blinded to cCT results. The TEE and TTE were performed with a Sparq ultrasound and EPIQ 7 echocardiography system (Philips Healthcare). The TTE was performed with a 1–5MHz probe and TEE with a multiplanar 2–7MHz probe (Philips Healthcare). All standard planes were examined and the resulting images stored digitally. The flow velocity in the LAA was determined in TEE using pulse-wave (PW) Doppler. An additional risk factor for a CES, spontaneous echo contrast (SEC), which is defined as a pattern of slow, swirling, and echogenic intra-arterial blood flow [10, 11] was reported when present at TEE or TTE. To determine the quality of SEC and to rule out thrombi, the LA and LAA were imaged in at least two planes. A thrombus in the LAA was defined as a circumscribed or solid, homogeneous, and echogenic mass with a nonmyocardial texture and the absence of blood flow velocity on PW Doppler.

### Statistical Analysis

Categorical baseline characteristics were expressed as numbers of patients and percentages. Owing to the lack of normal distribution, median values and interquartile ranges (IQR) were calculated and presented, rather than means and SDs.

Finally, echocardiographic results and confirmatory cCT were evaluated in a consensus reading by an experienced radiologist (P.L.) and experienced cardiologist (M. E.). Owing to the frequent absence of the reference standard (TEE) compared to cCT, we were not able to assess the sensitivity and specificity exactly and therefore only used a descriptive-exploratory style. All statistical analyses were performed using XLSTAT software (version 20.6 [Data Analysis and Statistical Solution for Microsoft Excel, Addinsoft, Paris, France]).

## Results

### Characteristics of Study Population

Between September 2018 and August 2019, 102 patients with suspected acute cardioembolic stroke due to large-vessel occlusion admitted to our tertiary stroke center were considered eligible for this retrospective study. Of these patients 60 were examined by using both cCT and echocardiographic approaches. Of note, 36 patients did not undergo echocardiography, either because of a lack of therapeutic consequences or treatment limitations to best supportive care, or owing to early demise of the patient. Demographic (age, sex), clinical (National Institutes of Health Stroke Scale [NIHSS]) and procedural characteristics (intravenous thrombolysis, endovascular thrombectomy [EVT], or echocardiography) are summarized in Table 1.

**Table 1:** Baseline demographic, clinical, and procedural characteristics

Number of patients	60
Male sex, *N* (%)	26 (43.3)
Age, years (IQR)	76 (72–82)
NIHSS	13 (5–20)
Intravenous thrombolysis, *N* (%)	22 (36.7)
Endovascular therapy, *N* (%)	35 (58.3)
Atrial fibrillation, *N* (%)	
*Yes*	26 (43.3)
*No*	32 (53.3)
*Unknown*	2 (3.3)

*IQR* interquartile range, *NIHSS* National Institutes of Health Stroke Scale

The median patient age was 76 years (IQR 71–82 years) and 34 patients were female (56.7%). Patients presented with a median initial NIHSS score of 13 (IQR 5–20), EVT was performed in 35 (58.3%) patients and/or intravenous lysis in 22 (36.7%) patients. Atrial fibrillation (AF) was unknown in 32 patients (53.3%).

### Performance and Diagnostic Yield of Delayed-phase Cardiac CT

Imaging findings are depicted in Table 2. Most of the patients presented occlusion of the middle cerebral artery (*n* = 47; 78.3%), with the right hemisphere overall being slightly more affected. In the remaining patients occlusion of the internal carotid artery (*n* = 4), posterior cerebral artery (*n* = 5), anterior cerebral artery (*n* = 2), vertebral artery (*n* = 1), or basilar artery (*n* = 1) was observed.

**Table 2:** Imaging findings

Number of patients	60
Hemisphere, *N* (%)	
*Right*	36 (60.0)
*Left*	23 (38.3)
*Bilateral*	1 (1.7)
Occlusion site, *N* (%)	
*ICA/ICA terminus*	4 (6.7)
*MCA*	47 (78.3)
*ACA*	2 (3.3)
*VA*	1 (1.7)
*BA*	1 (1.7)
*PCA*	5 (8.3)
Work-up echocardiography, days (IQR)	3 (2–5)
Echocardiography modality, *N* (%)	
TTE	50 (83.3)
TEE	10 (16.7)
Imaging quality CT, *N* (%)	
*Good*	42 (70.0)
*Moderate*	18 (30.0)
Cardiac CT findings, *N* (%)	
*Major CES*	10 (16.7)
*No major CES*	50 (83.3)
Echocardiography findings, *N* (%)	
*Major CES*	4 (6.7)
*No major CES*	56 (93.3)
DLP [mGy*cm] (IQR)	
*Complete CT*	1870 (1707–1969)
*Cardiac CT*	265 (219–344)

*ACA* anterior cerebral artery, *VA* vertebral artery, *BA* basilar artery, *PCA* posterior cerebral artery, *CES* cardioembolic sources, *CT* computed tomography, *ICA terminus* internal carotid artery terminus, *DLP* dose length product, *MCA* middle cerebral artery, *TEE* transesophageal echocardiography, *TTE* transthoracic echocardiography

**Fig. 1:**
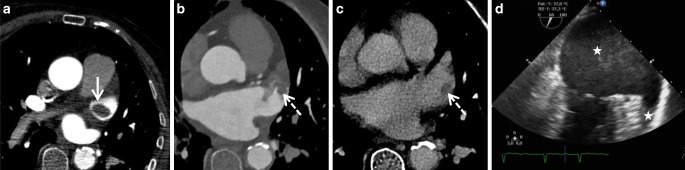
**a–c** Computed tomography in short-axis view of the heart and **d** transesophageal ecocardiography in four-chamber view. In this patient a thrombus decreased over time even without administering lysis therapy due to early cerebral infarct bleeding. In the initial examination, an oval contrast agent recess with a diameter of 24 mm in the sense of a thrombus can be seen (**a**, *white arrow*). At cardiac CT examination (**b**, **c**) 17 h after initial imaging, in the arterial (**b**) and venous phase (**c**) a smaller thrombus (10 mm, *white dotted arrows*) could be detected. Note the LAA circulatory disorder with triangular contrast agent sparing in the arterial phase (**b**). The TEE on day 7 shows spontaneous echo contrast (SEC) or smoke-like echo (*white stars*) without solid thrombus detection (**d**)

**Fig. 2:**
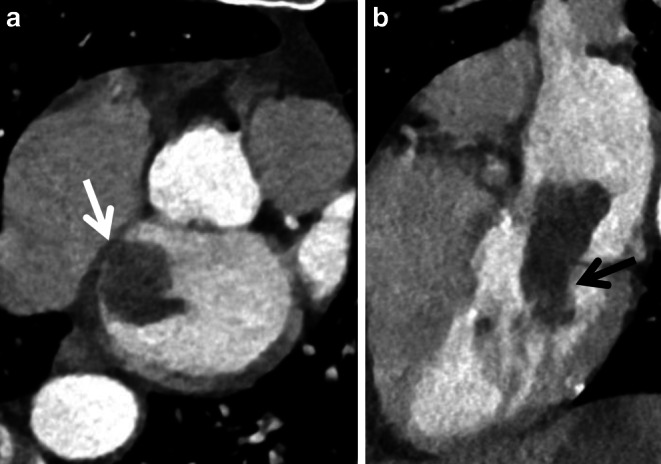
**a** Image section of computed tomography reconstructed in short-axis view and **b** in four-chamber view of the heart. In the short-axis view (**a**) a solid cardiac tumor mass can be seen in the left atrium (*white arrow*). The tumor has a relation to the septum, which is a typical feature of an atrial myxoma. In the four-chamber view (**b**) an extension via the mitral valve to the left ventricle can be seen (*black arrow*). After successful thrombectomy in a case of large-vessel occlusion, heart surgery was performed with subsequent histological confirmation of left atrial myxoma

**Fig. 3:**
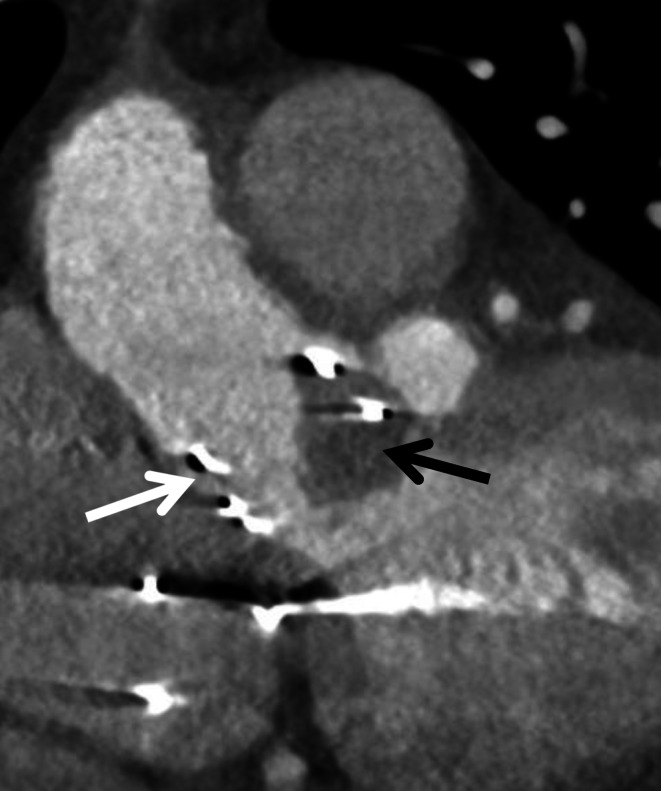
Image section of computed tomography in coronar view of the heart. Patient after previous transcatheter aortic valve implantation (TAVI) (*white arrow*) and acute thrombus detection on prosthesis valve struts (*black arrow*). This was regarded as the most likely cardiac embolic source responsible for acute ischemic stroke in this patient

**Fig. 4:**
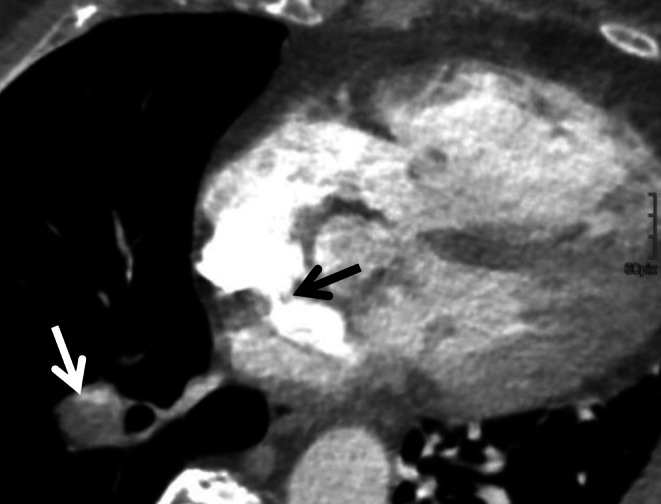
Image section of computed tomography in axial view of the heart. In this patient, a fulminant pulmonary embolism (*white arrow*) was observed in addition to the cerebral large-vessel occlusion. In cardiac imaging as part of the CS-CT protocol, a contrast jet in atrial septal defect with right-left shunt could be seen (*black arrow*), presumably as a cardiac cause of stroke due to additional paradoxical embolism. A TEE was not possible due to the poor general condition of the patient

In our study, the image quality of the cCT was predominantly rated as good (70%) or moderate (30%). In this study the assessment of the LAA was not limited due to inadequate contrast filling by flow artifacts, which is based on the delayed-phase cardiac acquisition with a preload of contrast agent due to CTA and CTP. In a total of 10/60 patients (16.7%), a major CES was detected by cCT, while most CES (6/10) were localized in LAA as thrombi (Fig. 1). In the other cases, cCT was able to visualize a ventricular thrombus, an atrial myxoma (Fig. 2), or valve thrombus deposits after transcatheter aortic valve implantation (TAVI) (Fig. 3), which were then interpreted as the most likely source of cardiac embolism. In one case an atrial septal defect with a severe right-left shunt could also be delineated in cCT and detected as the presumed cause of stroke due to paradoxical embolism (Fig. 4).

In none of the study patients was a contrast agent-associated side effect reported.

### Echocardiographic Examination

Of note, 36/102 (35.29%) of the AIS patients admitted to our stroke center did not undergo echocardiography. In 8 of the 36 patients in whom an echocardiographic examination was not performed, cCT also revealed a major CES. In 4 patients the examination could not be assessed due to the poor image quality. For the 60 patients, the median time interval between stroke onset and echocardiographic work-up was 3 days (IQR 2–5 days). A TEE was performed in 10 patients and TTE in 50. In 2 of the 50 patients a LAA thrombus could not be clearly ruled out by TEE due to high-grade SEC, but was definitively excluded in the initial cCT. In TTE the examiner always reported that anatomical and functional evaluation of the images was limited, especially at the atrial level. In a total of 4 patients (6.6%) a major CES was found by echocardiography. In two cases, a LAA thrombus was present and in one case each a ventricular thrombus and atrial myxoma was found.

### Diagnostic Yield of Cardiac CT Compared to Clinical Echocardiographic Work-up

Using the delayed-phase cardiac CT protocol, 10/60 (16.7%) major CES were detected but only revealed in 4 patients (6.7%) by echocardiography.

The cardiac thrombi observed by echocardiography in four patients were also detected by cCT. Overall, cCT demonstrated a substantially higher rate of detecting major CES than echocardiography (Table 3).

**Table 3:** Concordance between patients undergoing both examination of computed tomography and TTE (*N* = 50) or TEE (*N* = 10) for detection of major CES

	Major CES findings CS-CT (+)	Major CES findings CS-CT (−)	Total
CES findings echocardiography (+)	4	0	4
CES findings echocardiography (−)	6	50	56
Total	10	50	60

*CES* cardioembolic source, *CS-CT* cardiac stroke computed tomography

### Additional Radiation Exposure for Acquisition of Delayed-phase Cardiac CT

In our study, the total median dose length product (DLP) for the complete multimodal stroke and cCT examination resulted in 1870 mGy*cm (IQR 1707–1968 mGy*cm); for the cardiac part of the protocol the DLP amounted to 265 mGy*cm (IQR 219–344 mGy*cm). It is well known that arm position during a CT examination has a significant impact on image quality and radiation exposure [12]. Thus, the radiation dose is mainly due to the arm being positioned laterally to the body, which was the case in 60.0% (36/60) of patients.

## Discussion

Ischemic stroke is a leading cause of mortality and long-term disability worldwide and recurrence is considered to increase the incidence of severe disability and death [13, 14]. The common causes of AIS include arteriogenic or cardiogenic thrombi, plaque build-up, lacunar infarcts, and acute vessel dissection, or they remain cryptogenic. In 20–30% of all cases, an AIS is caused by a cardioembolism [15]. Cardiac causes of embolism have a poor prognosis, with higher in-hospital mortality and an increased short-term and long-term risk of recurrence [16]. The most common reasons for cardiac thrombus formation and cardiac embolism include primarily AF and atrial flutter with subsequent LAA thrombi, endocarditis, native valve diseases, and mechanical valve prothesis clotting, cardiomyopathies with remodeling and ventricular aneurysm formation, atrial myxoma as well as PFO, atrial septal defects and atrial septal aneurysm [17]. To prevent cardioembolic cerebral vessel occlusion, treatment with anticoagulants is urgently advised in patients with impaired left ventricular function or AF [18, 19].

While the number of stroke cases is continuously increasing, the average length of hospital stay is robustly decreasing [20]. Indeed, a cross-sectional survey on stroke units (SU) revealed a substantial heterogeneity among them concerning diagnostic work-up for detecting paroxysmal AF [21]. A systematic review [22] and economic evaluation described the use of TEE as comparatively low in older stroke patients and even the clinical work-up in our tertiary stroke center demonstrates that TEE is less frequently performed in patients with AIS than TTE, whereas TTE is known to be inferior in detecting CES [4]. European guidelines recommend at least 72 h of ECG monitoring for detecting AF, but this might not always transpire in clinical routine [23].

Especially in view of the fact that treatment failed in two clinical studies investigating oral anticoagulation in embolic stroke of undetermined source [24, 25], there is a vital need to assess potential improvements in stroke-related diagnostic procedures because the etiology of approximately 20–30% of all ischemic strokes remains unknown [21, 25]. The high percentage of unknown stroke etiology may be explained by low rates of TEE [5, 26] and insufficient duration of ECG monitoring [21]. Compared to TEE, cardiac CT and CTA of the aortic arch are less invasive and less time consuming and also have a growing potential for assuming a prominent diagnostic role in AIS.

### CT and Echocardiography in Stroke Patients

One-stop stroke CT is considered to be a noninvasive, fast procedure that is readily available. Moreover, CT is almost always feasible in a patient suffering from acute stroke and represents the primary imaging modality at most stroke centers. Studies have shown that in the context of stroke diagnosis cCT has good sensitivity and specificity for detecting sources of left cardiac emboli [9]. An additional main advantage of cCT is that the stroke etiology can already be verified during the initial emergency diagnostic work-up, thus potentially ensuring that adequate therapeutic decisions are made. In the clinical routine, a specialist for cardiac CT may not always be available at the time of initial imaging; however, the cCT could be evaluated later because it would most likely not change the acute stroke management. Indeed, the time to endovascular treatment should not be delayed under any circumstances.

Considering the TEE procedure as the reference standard in diagnosing a CES, however, TEE constitutes a semi-invasive procedure that is time consuming; moreover, it is not always feasible in an acute stroke setting. In addition, due to its invasive nature TEE can lead to complications [27] particularly in the upper digestive tract, the respiratory tract and in the cardiovascular system, although these complications are rare. The causes of TEE complications mainly reported include mechanical problems when inserting the probe, peri-interventional sedative drug administration associated with a significant drop in blood pressure, or inexperience of the examiner. Although TEE is considered very safe in the general population with a major complication rate of 0.02% [28], paradoxical air emboli during the evaluation for PFO have been reported [29] and the potential for periprocedural hypotension may be of greater consequence in a patient suffering from AIS [30]. These practical disadvantages of TEE can potentially delay implementing strategies to prevent recurrent stroke. Therefore, it is all the more important to demonstrate the diagnostic performance of early cardiac CT imaging to detect or exclude major CES. The excellent performance and diagnostic yield of cCT is based on continuous advances in CT technology, with increasingly faster acquisition times, improved image quality due to next-generation iterative algorithms, and the additional ability to reduce doses of radiation and contrast agent due to adapted protocols.

### Visualization of Major Cardioembolic Sources

In our study, most CES in cCT imaging were localized as thrombi in the LAA. In other cases, we were able to visualize an atrial myxoma or valve thrombus deposits after TAVI, which was then interpreted as the most likely source of cardiac embolism. The diagnostic yield in our patients, who were selected based on suspected acute cardioembolic stroke, may be higher than in a general ischemic stroke population.

Underlining the importance of early thrombus visualization, several studies have described the effect of thrombolysis, autolysis, and early anticoagulation on cardiac thrombi, which may result in thrombus fragmentation. Thus, although rarely, it may be difficult to detect the thrombus in time-delayed cardiac imaging. In an illustrative case in our cohort (Fig. 1), we were able to show that the cardiac thrombus initially defined in the LAA was already significantly smaller in the follow-up CT after 17 h and could no longer be detected in the TEE after a further period of 6 days. The thrombus had regressed without any lysis therapy due to an initial infarct bleeding. We thus assume that thrombus fragmentation also took place in other cases, in particular in patients who received additional thrombolysis. Nonetheless, a delay in the time interval between clinical stroke onset and cardiac thrombus/embolus imaging might therefore provide one explanation for the previously high numbers of cryptogenic strokes. This assumption is confirmed by the fact that no thrombus was missed using the cCT protocol, in comparison to echocardiography (TTE or TEE). In contrast, a cardiac thrombus was only found on the additional cCT in six patients in our study, which could have reduced the number of otherwise cryptogenic causes compared to echocardiographic assessment alone. Supporting this notion, we observed two clinical cases in which a LAA thrombus could not be verified by TEE due to high-grade SEC.

However, due to the additional need for human and logistic resources and given the fact that some patients were ineligible for transport or TEE, only 10 patients in our cohort had undergone TEE as reference standard. Thus, we may not have detected any possible misinterpretations of the cCT images.

### Visualization of Minor Cardioembolic Sources

It is known that in younger patients with AIS, TEE has the advantage of adequately assessing minor sources of paradoxical embolism such as PFO or conditions with relevant right-left shunt and thus additional radiation exposure can be avoided. In one case we could even visualize an atrial septal defect with a right-left shunt (minor CES) in the cCT and took this as the cause of a stroke due to a paradoxical embolism. In this respect, we expect that ongoing improvements in adapting contrast agent injection protocols and the additional impact of spectral-based CT systems will help overcome such disadvantages of CT in the future.

### Advantages and Disadvantages of the Delayed-Phase Cardiac CT Protocol

The well-known limitations of cardiac CT for accurately assessing LAA thrombus have been described in terms of possibly delayed LAA filling. Studies have shown that sensitivity and specificity and positive and negative predictive values alike can be increased if the cardiac examination is delayed until a contrast agent has been administered [28]. Slow-flow artifacts in areas such as the LAA can be avoided by giving a prebolus of a contrast agent. Our cCT protocol involved two steps: CTA and CTP acquisition as the first and ECG-gated cardiac imaging as the second step. This imaging procedure employed a triphasic injection protocol with only 20 ml more contrast agent than given in the standard stroke CT protocol and resulted in an overall good image quality. Image quality was improved by additionally optimizing and concomitantly reducing contrast agent dosages in CTA and CTP and optimally timing our CTA acquisition based on the perfusion CT series used as bolus test. This combined acquisition shortens the examination time and reduces the contrast agent dose. Moreover, during delayed-phase scanning contrast agent and blood become completely mixed, resulting in a kind of equilibrium phase. By using this technique, a thrombus can be easily distinguished from blood stasis by different contrast agent attenuations. In their study, Taina et al. [29] even demonstrated that cardiac CT has a higher sensitivity and specificity than TEE, especially in patients with a reported SEC. Given the short acquisition time of modern CT systems, cardiac CT can be easily implemented in the acute stroke imaging protocol without delaying the acute stroke work-up [30].

However, the additional radiation exposure as well as contrast agent administration constitute significant disadvantages when a separate, ECG-triggered cardiac CTA is added to the standard stroke imaging protocol [31]. Our results demonstrate that the cCT protocol provides an advantage in the early diagnosis of major CES in terms of time to diagnosis and diagnostic accuracy. In contrast, the risk of radiation-induced diseases and the loss of life expectancy from radiation-induced diseases are lower in this older patient group since life expectancy is mostly lower in these patients due to comorbid conditions, chronic disease, and advanced age.

### Study Limitations

Our study has several limitations, which are mostly inherent to the retrospective nature of this study. This study design may lead to a selection bias, as it is unknown why some patients underwent cCT but did not undergo echocardiographic work-up. We cannot determine whether imaging findings impacted patient management at the time. Secondly, only a minority of patients underwent TEE; consequently, it was not possible to assess the sensitivity and specificity between cCT and TEE as reference standard especially for assessing LAA thrombus. Furthermore, the time interval between cCT and echocardiography of 3 days (IQR 2–5 days) represents clinical reality for work-up but we cannot rule out that a thrombus formed or resolved during this period. Additionally, we did not perform contrast agent-enhanced echocardiography to assess LAA thrombus and SEC and no interobserver agreement measurement was performed.

However, considering the study limitations our findings serve as a proof of concept: major CES can be detected on extended stroke CT with delayed-phase cardiac CT before they are potentially dissolved by thrombolysis or flushed into the circulation. We looked at patients with known large vessel occlusion suspected of being the source of cardioembolic stroke, which increased the pretest probability for cardiac thrombi; however, the greatest clinical benefit of this method would be expected in patients with cryptogenic stroke, where detecting a cardiac thrombus has profound implications for acute treatment (early anticoagulation and follow-up imaging) and secondary prevention (long-term anticoagulation). Prospective multicenter studies should explore this idea with delayed-phase cardiac CT in the future. In the long run, a one-stop stroke CT multimodal CT program could be developed to optimize emergency stroke diagnostics. This novel approach is promising. Future prospective studies should also compare the diagnostic yield of acute cardiac CT with echocardiography and monitor the impact of cardiac CT findings on patient management and outcome and, in addition, address the costs of different modalities and availability in community hospitals.

Furthermore, larger trials may demonstrate whether implementing cardiac CT in acute stroke imaging protocols ensures that minor CES such as PFO will be detected. In addition, attempts should be made to further reduce contrast agent dosages and radiation exposure. In this respect, new reconstruction algorithms and spectral CT imaging have the potential to improve the contrast image quality and make it possible to detect cardiac thrombus without ECG gating and without additional contrast agent [9, 31].

## Conclusion

This study represents a starting point for evaluating diagnostic performance of a delayed-phase cardiac CT added to the initial multimodal stroke CT in patients with suspected acute cardioembolic stroke. These preliminary results demonstrate the potential yield for detecting major CES, and therefore could accelerate decision-making to prevent recurrent stroke. Consequently, larger studies with TEE as reference standard and also compared to TTE are needed to confirm these results. Cardiac CT does not replace a complete cardiac assessment, however, and a dedicated cardiac examination may still be indicated.
